# Treatment Strategies and Metabolic Pathway Regulation in Urothelial Cell Carcinoma: A Comprehensive Review

**DOI:** 10.3390/ijms21238993

**Published:** 2020-11-26

**Authors:** Huang-Yu Yang, Chao-Yi Wu, Jia-Jin Chen, Tao-Han Lee

**Affiliations:** 1 Department of Nephrology, Chang Gung Memorial Hospital, College of Medicine, Chang Gung University, Taoyuan 33305, Taiwan; Raymond110234@hotmail.com; 2 Department of Health Policy and Management, Johns Hopkins Bloomberg School of Public Health, Baltimore, MD 21205, USA; 3 Division of Allergy, Asthma, and Rheumatology, Department of Pediatrics, Chang Gung Memorial Hospital, College of Medicine, Chang Gung University, Taoyuan 33303, Taiwan; joywucgu@cgmh.org.tw

**Keywords:** urothelial carcinoma, immune checkpoint inhibitors, immunotherapy, tumor microenvironment, metabolic pathway

## Abstract

For a long time, cisplatin-based chemotherapy had been viewed as first-line chemotherapy for advanced and metastatic urothelial carcinoma (UC). However, many patients with UC had been classified as cisplatin-ineligible who can only receive alternative chemotherapy with poor treatment response, and the vast majority of the cisplatin-eligible patients eventually progressed, even those with objective response with cisplatin-based chemotherapy initially. By understanding tumor immunology in UC, immune checkpoint inhibitors, targeting on programmed death 1 (PD-1) and cytotoxic T lymphocyte-associated antigen 4 (CTLA-4) pathways, had been proven as first-line treatment for cisplatin-ineligible metastatic UC and as second-line treatment for patients with platinum-refractory metastatic UC by the U.S Food and Drug Administration (FDA). In 2020, JAVEIN bladder 100 further reported that PD-L1 inhibitors showed benefits on prolonged survival and progression-free survival as maintenance therapy. Besides targeting on immune checkpoint, manipulation of the tumor microenvironment by metabolic pathways intervention, including inhibition on tumor glycolysis, lactate accumulation and exogenous glutamine uptake, had been investigated in the past few years. In this comprehensive review, we start by introducing traditional chemotherapy of UC, and then we summarize current evidences supporting the use of immune checkpoint inhibitors and highlight ongoing clinical trials. Lastly, we reviewed the tumor metabolic characteristic and the anti-tumor treatments targeting on metabolic pathways.

## 1. Introduction

Bladder cancer is the most common malignancy involving the urinary system and a common malignancy worldwide. Since the first efficacy immunotherapy for non-muscle invasive bladder cancer, bacillus Calmette-Guérin (BCG), was established by Morales and associates in 1976 [1], no immunotherapy has been approved and cisplatin-based chemotherapy has been viewed as first-line chemotherapy for advanced and metastatic urothelial carcinomas (UCs) for a long time. With the better understanding of cancer immunology, immune checkpoint inhibitors and metabolic pathway intervention have been investigated to break this therapeutic stalemate in the past few years [2,3]. In this review, we introduce traditional chemotherapy of UCs and summarize current evidences supporting the use of immune checkpoint inhibitors in advanced urinary system malignancy. Lastly, we review the new approaches of immune oncology via metabolic pathways.

## 2. Epidemiology and Pathogenesis of Urothelial Carcinoma

The American Cancer Society reported about 80,470 new cases of bladder cancer and about 17,670 deaths due to bladder cancer in 2019 [4]. Among the various histological types of bladder cancers, urothelial carcinoma of the bladder (UCB) is the most common and is referred to as upper tract urothelial carcinoma (UTUC) when its involvement site changes to the renal pelvis and ureter. Although UTUC is less common than UCB, it usually shows more invasive staging at diagnosis. Tobacco smoking is a well-known risk factor for both UCB and UTUC and is responsible for more than 50% of all UCs. In addition to smoking, aristolochic acid (AA) consumption has been identified as an important environmental risk factor for UCs in the past few years. AA is a common component in Chinese medicines and some weight loss regimens in Western countries and has been linked to Chinese herbal nephropathy and Balkan endemic nephropathy. It generates AA-derived DNA adducts and is accompanied by a high frequency of A:T pair mutations of the *TP53* gene [5,6,7]. According to a previous molecular epidemiology report, the ratio of UTUC to total UCs is significantly higher in endemic areas and more than half of these UTUC cases are possibly associated with AA exposure [6]. Before the development of effective chemotherapy strategies, UC was a lethal disease, with a median survival of 3–6 months, and nearly all patients died from progression to locally advanced cancer or metastatic disease [8]. The survival outcomes of UC were greatly improved since the use of cisplatin-based chemotherapy as first-line therapy for both locally advanced and metastatic UC; however, more than 50% of patients were considered ineligible for cisplatin-based chemotherapy due to compromised performance status, renal failure, or heart failure. Most cisplatin-ineligible patients had no choice but to receive alternative regimens that have less efficacy. This situation has changed in the past few years with the use of immune checkpoint inhibitors (ICIs) in the management of UCs. ICIs have shown positive results as first-line treatment in cisplatin-ineligible patients and second-line treatment in patients with platinum-refractory metastatic UC. In this comprehensive review, we have summarized the treatment strategies in UC, including traditional chemotherapy and immunotherapy, and provided new evidence regarding metabolic interventions in the management of malignancies.

## 3. Chemotherapy in Urothelial Carcinoma

For non-muscle-invasive UCB (stage Ta, T1, and Tis) or low-risk UTUC, conservative treatment including transurethral resection, kidney-sparing surgery, and intravesical therapy appears to be a rational choice considering the importance of preserving bladder and renal function [9,10]. However, approximately 25% of patients will progress to muscle-invasive disease or develop metastatic disease after initial conservative therapy, leading to the development of second primary tumors along the urothelium in the genitourinary tract, including the renal pelvis, ureters, urethra, and bladder. When the disease progresses to advanced stages or a metastatic phase, systemic chemotherapy becomes the standard approach, and cisplatin-based chemotherapy is the initial choice of therapy for such patients. Gemcitabine plus cisplatin (GC) and dose-dense methotrexate with vinblastine, doxorubicin, and cisplatin (M-VAC) are the common chemotherapy regimens for metastatic UC and have shown similar survival benefits, with a median overall survival of approximately 13.8 and 14.8 months, respectively [8,11,12]. Although M-VAC therapy has a response rate of 72% for metastatic disease, its high toxicity and low tolerability have been a major concern. Conversely, the GC regimen appears to have a better safety profile and higher tolerability than the M-VAC regimen, but in a previous report, nearly 30% of patients experienced anemia and 50% of patients developed thrombocytopenia after GC therapy [8,12,13]. In 2011, Galsky et al. defined that cisplatin-ineligible patients have one of the following characteristics: Eastern Cooperative Oncology Group (ECOG) performance status ≥2, neuropathy grade ≥2 or hearing loss, New York Heart Association Classification III heart failure or higher, or creatinine clearance <60 mL/min [14,15,16,17,18]. Subsequently, this criterion became an important index to evaluate whether cisplatin-based chemotherapy is suitable for patients. However, radical nephroureterectomy with bladder cuff excision with or without template preservation is the standard of care for high-risk UTUC; however, renal function preservation is more challenging in radical nephroureterectomy and patients undergoing this procedure are often unsuitable for cisplatin-based chemotherapy. In a study by Kaag et al., the mean estimated glomerular filtration rate (eGFR) declined significantly after nephroureterectomy: the mean eGFR decreased by 24% compared to the preoperative level. Further, patients eligible for chemotherapy decreased from 49% to 19% after operation using eGFR of 60 mL/min per 1.73 m^2^ as the cutoff value [19].

For cisplatin-ineligible patients, carboplatin-based treatment is considered an alternative; however, carboplatin-based chemotherapy shows disappointing results compared to the cisplatin-based treatment. A study reported that carboplatin-based treatment had a significantly lower percentage of overall response and complete response than a cisplatin-based regimen [20,21]. Considering the poor response shown by alternative chemotherapy regimens in cisplatin-ineligible patients and that a vast majority of cisplatin-eligible patients eventually progress despite objective response in initial cisplatin-based chemotherapy, novel therapy agents had been sought for the treatment of locally advanced and metastatic UC, and ICIs have become the most promising alternative therapy in the past few years. 

## 4. Immunotherapy in Urothelial Carcinoma

By understanding the immune system and interaction between immune cells and tumor cells, immunotherapy for cancer patients has tremendously expanded in the past decades. In normal physiology, the interaction between antigen-presenting cells (APCs) and T cells is well controlled by a complex and rigorous system, the immune checkpoint system. This system suppresses the adaptive immune response in a timely manner to prevent incorrect or prolonged T cell activation. The co-stimulatory or inhibitory proteins on APCs are key components of the immune checkpoint system that determine the fate of T cells after antigen presentation. For example, when protein CD80/86 on APCs bind to CD28 protein on T cells, a stimulatory response is triggered, leading to T cell proliferation. In contrast, the inhibition cascade is activated when the same protein, CD80/86, binds to the cytotoxic T lymphocyte-associated antigen 4 (CTLA-4) or when the programmed death ligand 1 (PD-L1) on APCs binds to programmed death 1 (PD-1) protein on the T cells. Based on the current understanding of cancer immunity, cancer cells may evade the anti-tumor response by exploiting these immune checkpoint pathways and creating a tumor microenvironment (TME) that ultimately impairs immune cell activation and function. In traditional chemotherapy, the cytotoxic effect is mostly based on blocking the cell cycle via interrupting DNA replication, inhibiting metabolic function, which not only kills the cancer cells but also influences normal cells’ reproduction. But ICIs focus particularity on rejuvenating T cell anti-tumor response by using antibodies or recombinant ligands to block malignant cell-induced inhibitory pathways. Through targeting the antigen-dependent pathway between APCs and T cells, ICIs intensify immune response, which had more specific targeting on cancer cells and had lesser influence on healthy cells compared to the traditional chemotherapy [22,23,24,25]. 

The antagonistic antibodies of CTLA-4 (ipilimumab and tremelimumab) and PD-1 receptor (nivolumab, pembrolizumab, and pidilizumab) or its ligand PD-L1 (atezolizumab, avelumab, and durvalumab) are two classes of ICIs approved by the United States Food and Drug Administration (FDA). Tissue-based assays in various studies suggest that tumors with larger sizes, aggressive behaviors, and poor outcomes are associated with PD-L1 expression [23,26,27]. Furthermore, recent data suggested that ICIs are more active in tumors with high mutation rates than those with lower mutation rates [25,28,29,30,31]. Garon and associates had reported that non-small cell lung cancer patients whose membranous PD-L1 expression was less than 50% had shorter progression-free and overall survival after ICIs treatment comparing to higher PD-L1 expression patients [28]. In human gastrointestinal cancers, Tran et al. not only established that patients with higher immunogenic mutation showed longer tumor regression time after ICIs treatment but also pointed out that the neo-epitope T-cell response had relatively low to moderate mutation burdens, which are not susceptible to current immune checkpoint modulators [29]. Higher mutational load, which included mismatch, mircrostatellite instability, or DNA methylation, indicated better response to immune checkpoint blockade and was concluded in the review of cancer treatment with immune checkpoint inhibitor by Thallinger et al. [25]. These studies propose that ICI treatment would be more suitable in malignancies with a high somatic mutation rate because abundant tumor-specific neo-antigens might induce a rigorous immune response post T cell rejuvenation by ICIs. Based on these findings, ICIs appear to be an excellent choice for advanced and metastatic UCs, which not only exhibit aggressive clinical behavior but also are well-known highly antigenic malignancies harboring the fourth highest mutation rate among all cancers [32]. 

### 4.1. Immunotherapy: PD-1 Signal Inhibitor

In 1999, Honjo et al. demonstrated that receptor PD-1 is an immune checkpoint based on the finding that PD-1 knockout mice develop autoimmune diseases. The ligands of PD-1, PD-L1, and PD-L2 were identified in 2000 and 2001, respectively. By understanding that tumors can escape host immune surveillance by expressing PD-L1, blocking the PD-1/PD-L1 signaling pathway to improve the outcomes in patients with malignancies seems rational [33,34,35]. In 2006, the first clinical study using PD-1 signal inhibitor (MDX-1106) against treatment-resistant solid tumors showed improved anti-tumor efficacy and high tolerability [36]. To date, there are nine types of PD-1 signal inhibitors from eight pharmaceutical companies, and more than 500 clinical trials are examining their efficacy in different types of solid and hematological malignancies. 

Among these nine types, the FDA has approved two PD-1 signal inhibitors as first-line therapy and five PD-1 signal inhibitors as second-line therapy for locally advanced and metastatic UC. Atezolizumab and pembrolizumab were both approved as first-line therapies for cisplatin-ineligible or platinum-ineligible patients with PD-L1-positive advanced or metastatic UC. Atezolizumab, a humanized engineered immunoglobulin G1 monoclonal antibody that inhibits binding to PD-L1, was first used as first-line treatment for cisplatin-ineligible patients with metastatic UC in the IMVigor 210 trial in 2016. Most cisplatin-ineligible patients had renal impairment and low ECOG performance and some had a history of hearing impairment and peripheral neuropathy. In this trial, atezolizumab showed promising response durability and overall survival with low clinically relevant toxicities in metastatic UC [37]. Almost at the same time, the anti-PD-1 antibody pembrolizumab was investigated for cisplatin-ineligible patients with metastatic UC in the KEYNOTE-052 trial, which showed a 27% complete response rate with acceptable tolerability. The PD-L1 expression level was examined in the KEYNOTE-052 trial, and prolonged median overall survival was observed in patients with PD-L1 combined positive scores ≥10 [38]. 

Following the failure of platinum-based therapy in patients with advanced or metastatic UC, the FDA approved atezolizumab, avelumab, durvalumab, nivolumab, and pembrolizumab as second-line treatment. IMVigor210 reported that atezolizumab showed promising results with respect to overall survival and progression-free survival in platinum-refractory metastatic UC, and the result was even better in patients with higher levels of PD-L1-positive immune cells [30]. IMVigor211, a phase III clinical trial, compared atezolizumab to the investigator’s choice of therapy (taxanes or vinflunine) for platinum-refractory metastatic UC. Despite failing to meet the primary endpoint of the study, the exploratory analysis showed durable response and survival benefits of atezolizumab in the intension to treat papulation [39]. Furthermore, pembrolizumab was reported to have a prolonged overall survival and lower treatment-related adverse events rate as compared to chemotherapy (paclitaxel, docetaxel, or vinflunine) for platinum-refractory advanced or metastatic UC in the KEYNOTE-045 trial. In this study, pembrolizumab prolonged overall survival regardless of PD-L1 status [40]. Durvalumab, a selective, high-affinity, engineered human monoclonal antibody blocking PD-L1 binding to PD-1, was used in patients with metastatic UC who progressed after the failure of platinum-based chemotherapy in the NCT01693562 clinical trial. In this single-arm phase I/II trial, durvalumab showed favorable results in treatment response rate and overall survival regardless of PD-L1 expression level. Nivolumab, a human monoclonal antibody targeting PD-1, was first reported to have a durable clinical response and manageable safety profile in platinum-refractory advanced or metastatic UC in the CheckMate 032 trial, and significant improvement in overall survival among different PD-L1 expression levels was then observed in the CheckMate 275 study [41,42]. In the JAVELIN solid tumor study, Avelumab, a human immunoglobin G1 anti-PD-L1 antibody, showed anti-tumor activity in platinum-refractory or cisplatin-ineligible patients, with 40% objective response rate in PD-L1-positive tumor cells in >5% of patients and 9% in PD-L1-positive cells in <5% of patients [43]. 

Although PD-1 signaling inhibitors showed promising results as monotherapy, a low long-term durable objective response rate and high relapse rate warrants alternative approaches for metastatic UC. Previous studies had revealed that the chemotherapy can induce immunogenic cell death (ICD), a regulated cell death that enables the initiation of adaptive immunity in immunocompetent syngeneic hosts, which leads to interferon γ signaling increase and upregulation of major histocompatibility complex (MHC) class I molecules, releasing adenosine triphosphate (ATP) and damage-associated molecular patterns (DAMPs), influencing transcriptomic perturbations and the accumulation of genetic and epigenetic defects [44,45,46]. These changes turned immunologically ‘cold’ tumors to ‘hot’ lesions with APCs and T cells abundantly infiltrated. A variety of animal studies had proven that the conventional chemotherapeutic agents can promote on-target immunostimulatory effects by increasing the antigenicity or adjuvanticity of cancer cells [44,47,48,49,50,51,52,53]. Based on these findings, several clinical trials had attempted to combine chemotherapy and ICIs in treating advanced and metastatic UCs. In IMVigor310, a double-blind phase III trial, combination therapy with atezolizumab plus platinum-based chemotherapy showed significant prolongation of progression-free survival and enhanced interim overall survival and complete response rate [54]. However, another phase III trial, KEYNOTE-361, which compared pembrolizumab with chemotherapy and pembrolizumab alone, failed to meet the primary endpoint, and showed that combination therapy was not superior to pembrolizumab monotherapy with respect to both overall survival and progression-free survival [55]. Currently, ongoing trials are investigating the efficacy of a combination of systemic chemotherapy and checkpoint inhibitors in locally advanced and metastatic UC. Future application of these potential regimens will be determined by the results of these clinical trials.

Besides being the first-line treatment for cisplatin-ineligible or platinum-ineligible metastatic UC and second-line treatment for patients with platinum-refractory metastatic UC, recent reports have revealed that immune checkpoint therapy may also play a role in maintenance therapy. A recently published phase III trial, JAVEIN bladder 100, reported that using avelumab, an anti-PD-L1, as maintenance therapy for patients with locally advanced or metastatic UCB who experienced an objective response or stable disease after four to six cycles of GC regimen, prolonged overall survival and progression-free survival compared to the best supportive care. This encouraging result was observed across all prespecified subgroups regardless of PD-L1 expression status [56,57]. Based on these data, in 2020, Avelumab was approved by the FDA for maintenance therapy in metastatic UC not progressed after initial platinum-based chemotherapy. The FDA proved indication and therapeutic regimen of PD-1 signal inhibitor were summarized in Table 1.

### 4.2. Immunotherapy: CTLA-4 Pathway Inhibitor

In 1988, CTLA-4 was first recognized as a critical molecule in maintaining T cell homeostasis and tolerance by competing with CD28 for CD80/86 and downregulating T cell receptor (TCR) signaling. The TCR signaling pathway activated by CD28-CD80/86 promotes cytokine interleukin-2 (IL-2) production and leads T cells into the cell cycle entry, T helper-cell differentiation, and immunoglobulin isotype switching; therefore, CTLA-4 induces T cell anergy by blocking these pathways [58,59,60]. One theory suggests that the poor immunogenicity of many malignancies may be because these tumors cannot provide a signal strong enough for CD28-medicated co-stimulation, which is necessary to fully activate T cells. Based on this theory, Leach et al. reported that the administration of CTLA-4 antibodies can induce an effective anti-tumor response by blocking the inhibitory effects of CTLA-4 [15,61,62]. To date, there are two CTLA-4 inhibitors being examined for their efficacy in various malignancies in several clinical trials.

Tremelimumab, a monoclonal antibody against CTLA-4, has been used as monotherapy for patients with platinum-refractory locally advanced or metastatic UC in the NCT02527434 trial. In this phase II trial, 32 patients with metastatic UC were enrolled and 750 mg tremelimumab was given via intravenous infusion every four weeks for seven cycles and then every 12 weeks for two additional cycles, which resulted in better clinical activity with an objective response rate of 18.8% [63]. There seems to be similar clinical efficacy between CTLA-4 blocking with tremelimumab and PD-1/PD-L1 inhibitor in platinum-refractory UC; however, patients tend to experience more side effects with CTLA-4 inhibition according to the American Association for Cancer Research [64]. Further validation from studies with larger numbers of patients is needed before clinical application.

Considering that ICIs targeting the CTLA-4 and PD-1/PD-L1 pathways have distinct mechanisms of T cell response, combination therapies targeting both these pathways to manipulate different phases of immune responses may improve antitumor efficacy [58,65,66,67]. Based on this theory, synergistic immunotherapeutic activity of combined inhibition of the CTLA-4 and PD-1/PD-L1 pathways were tested. The CheckMate 032 trial compared nivolumab monotherapy to combination therapy with nivolumab and ipilimumab in platinum-refractory patients, and the combination therapy group was further divided by different ICI dosages. Patients received either 3 mg/kg nivolumab plus 1 mg/kg ipilimumab every three weeks for four doses, followed by 3 mg/kg nivolumab monotherapy every two weeks, or 1 mg/kg nivolumab plus 3 mg/kg ipilimumab every three weeks for four doses, followed by 3 mg/kg nivolumab monotherapy every two weeks. The combination therapy group showed a higher objective response rate to nivolumab monotherapy, and within the combination therapy group, patients who received higher ipilimumab doses showed better objective response rates and complete response rates. No significant toxicities induced by combination therapy were reported in this study [68]. Based on these results, a phase III trial, CheckMate 901 (NCT03036098), comparing nivolumab with or without ipilimumab with cisplatin or carboplatin-based chemotherapy for metastatic UC in the first-line setting is ongoing. Furthermore, therapy with durvalumab alone or in combination with tremelimumab is being investigated in the NCT02527434 phase II trial for evaluating its safety and efficacy, and duravalumab and tremelimumab combination therapy for patients with stage IV bladder cancer is being investigated in the NCT02516241 phase III trial.

### 4.3. Immunotherapy: Limitations and Adverse Effects

Although ICIs had shown encouraging results no matter whether treating first-line treatment, second-line treatment, or maintenance therapy in advanced and metastatic UCs, there were still some limitations in ICIs treatment. Immune-related adverse events (IRAEs) had been noticed in a considerable percentage of patients receiving immune checkpoint inhibition therapy; these adverse effects, which mainly involve the gut, skin, liver, lung, and endocrine, were induced by over-stimulation of immune reactivity or generated autoimmune phenomena [69]. According to a metastasis analysis published by Velasco et al., ICIs were associated with more colitis, aspartate aminotransferase (AST) elevation, and pneumonitis compared to a non-ICIs treatment group based on 21 randomized phase II/III immunotherapy trials [70]. Despite the fact that severe IRAEs had been reported in patients treated with CTLA4 and PD-1/PD-L1 pathway inhibitors, less than 1% of all patients had ICI-related fatal events across all clinical trials [70]. There were many issues in ICI-related IRAEs which remain unclear and unresolved. Every symptom has to be suspected as a sign of IRAEs in patients under ICIs treatment, so we should inform patients to contact their treatment group once a possible adverse effect occurs. Doctors who prescribe ICIs should remain highly alert once patients report new symptoms during ICIs treatment, because early diagnosis and intervention can prevent IRAEs progressing from grades 1–2 to grades 4–5 toxicities [24,69,71].

## 5. Metabolic Intervention as Cancer Treatment

ICIs showed promising clinical activity and durable side effects in locally advanced or metastatic UC, as mentioned above. The overall response rate was still around 30%, according to a previous report [72]. Several studies have revealed a lack of correlation between T cell infiltration in solid tumors, response to immunotherapy, and density of immunogenic antigens, suggesting that there are antigen-independent factors that influence the anti-tumor response [73,74,75,76]. Based on the experimental and clinical evidence, the tumor microenvironment (TME) might be an antigen-independent factor that influences immune surveillance. TME is characterized by a complex interplay between tumor cells and their surrounding neighbors, including stromal cells, extracellular matrix, adipocytes, and mesenchymal stem cells. TME compromises the function and the fate of tumor-infiltrating immune cells by creating a three-dimensional structure favoring immunological tolerance and reducing anti-tumor efficacy, even with immunotherapeutic intervention. All the components within TME, including low pH, hypoxia, nutrient-limiting metabolic competition, and nitric oxide (NO) production work together to impair T cell function [3,22,77,78,79,80]. 

In 1923, Warburg et al. observed that tumor cells exhibit high glucose uptake and favor glycolysis as an energy supplement, even with sufficient oxygen, which was coined as the Warburg effect [81,82,83,84]. However, glycolysis is also required by T cells, macrophages, and APCs; therefore, competitive TME impairs these immune cells by limiting their glycolytic capacity. Sirtuin 6 (SIRT6), a nicotinamide adenine dinucleotide (NAD+)-dependent deacetylase, is involved in glucose homeostasis as an epigenetic regulator. SIRT6 deficiency stimulates hypoxia-inducible factor 1α (HIF1α) activity, which increases the expression of glycolysis-related genes, such as lactate dehydrogenase (*LDH*), glucose transporters (*GLUTs*), and pyruvate dehydrogenase kinases (*PDKs*) [85,86,87]. Several studies have revealed that various human cancers, especially pancreatic and colorectal cancer, are SIRT6-deficient, and restoring SIRT6 activity might be a promising therapeutic strategy in these cases [88,89,90]. Ellagic acid, a common polyphenol in berries, had been reported to have an anti-proliferative effect on human colon adenocarcinoma cells by inhibiting glycolysis via increasing the deacetylase activity of SIRT6 [91,92,93]. This suggests that targeting SIRT6 activity might be a new insight into the development of anti-cancer therapies. According to the Warburg effect, tumor cells favor glycolysis, which leads to excessive lactate formation even under sufficient oxygen. Lactate, the main metabolite of tumor cells and cancer-associated fibroblasts, is largely accumulated in the TME and has been seen as one of the key components driving immunosuppression and favoring tumor growth [94,95,96,97]. Growing evidence indicates that the proton-coupled lactate efflux from cancer cells or stromal cells preserves the acidic phenotype and increases tumor progression by modulating the TME, which includes cell invasion, angiogenesis, survival signaling, and escaping immune surveillance.

An in vitro study reported that human and mouse tumor-specific CD8^+^ cells reduce cytolytic activity and cytokine production when extracellular pH is lower than 6.0–6.5 [98,99]. By understanding these mechanisms, molecule targets for lactate transporters, SLC16A1 or SLC16A7, to reduce lactate levels in tumors have been discovered. AZ3965 and α-cyano-4-hydroxycinnamate, small molecules blocking lactate transporters, have been reported to be successful in several types of malignancies including Burkitt lymphoma, breast cancer, small-cell lung cancer, and glioblastoma. Another therapeutic approach is to block the conversion of pyruvate to lactate by inhibiting lactate dehydrogenase A (LDHA) [100,101]. Negative correlations between LDHA expression and T cell activation markers were noted in mice tumor models, and further studies revealed that LDHA targeting therapy can re-activate T cell-mediated and natural killer cell-mediated immune surveillance [102]. N-hydroxyindole and galloflavin, LDH inhibitors, have shown encouraging efficacy on reducing cancer cell growth and inducing apoptosis [103,104].

In addition to favoring glycolysis, many cancer cells depend on an exogenous supply of glutamine. Glutamine, a non-essential amino acid in mammalian cells, is an important fuel for the tricarboxylic acid cycle, an important source of reduced nitrogen for biosynthesis, and a precursor of nucleotide and lipid synthesis. Although most mammalian cells can synthesize glutamine de novo via glutamine synthetase (GLUL), several types of cancer cells only express low levels of GLUL and need to depend on exogenous glutamine supplementation, which can be catabolized in the mitochondria via glutaminase [105,106,107,108]. Glutamine metabolism is a key component not only for tumor survival but also for regulating the balance between effector T cells and T regulatory cells. Studies have revealed that reduction of glutamine levels in culture media reduced mammalian target of rapamycin complex 1 (mTORC1) activity and coincided with effector T cell defects, suggesting that the glutamine-depleted TME would suppress anti-tumor immunity [3,109,110,111,112]. In the recent years, by understanding the high demand of glutamine in cancer cells and its regulatory effect on the immune system, the glutamine antagonist 6-diazo-5-oxo-L-norleucine has been re-evaluated as an anti-cancer therapy, especially in glutamine-dependent tumors [113]. Furthermore, CB-839, a potent selective and orally bioavailable inhibitor of glutaminase, showed anti-proliferative activity on tumor cell lines and is being evaluated in clinical trials for the treatment of glutamine-dependent malignancies [114,115,116]. The metabolic characteristic of dependence on exogenous supplement of glutamine in tumors suggests that surrounding neighbors might play a key role in glutamine supplement. As the cancer grows, they recruit stromal cells as part of the TME. These reactive stromal cells co-evolve and continually interact with cancer cells, becoming an integral part of their physiology and indispensable for their survival. In 2016, Yang et al. identified that reactive stromal cells are reprogrammed through an upregulated glutamine anabolic pathway, which allows them to harness carbon and nitrogen from non-canonical sources to synthesize glutamine in nutrient-deprived TME and become the exogenous glutamine source for tumor development. In a mouse tumor model, co-targeting glutamine synthetase in stromal cells and glutaminase in cancer cells can reduce tumor size, weight, and prevent metastasis [106]. 

**Figure 1:**
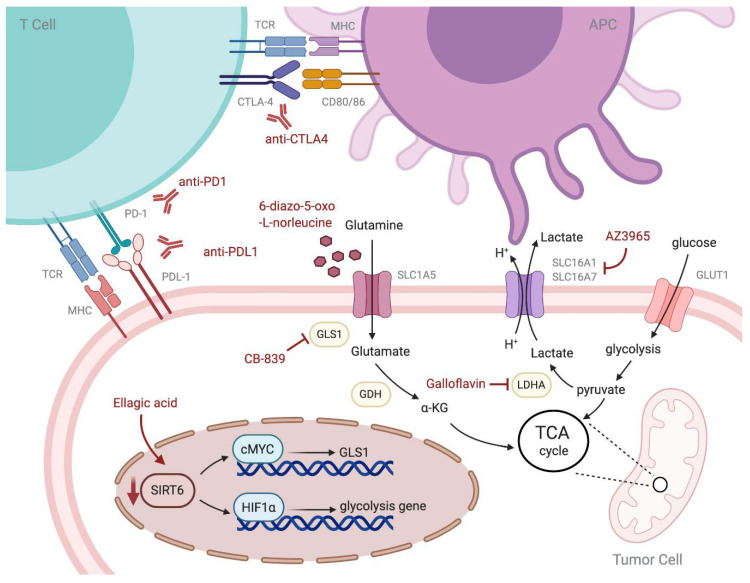
The immune interaction and metabolic character of tumor cells and the mechanism of immune checkpoint inhibitor and metabolic intervention. APC: antigen-presenting cell; α-KG: α-Ketoglutaric acid; GLUT1: glucose transporter 1; HIF1α: hypoxia-inducible factor 1α; SIRT6: Sirtuin 6; TCA cycle: tricarboxylic acid cycle; GLS1: glutaminase; GDH: glutamate dehydrogenase; LDHA: lactate dehydrogenase A; PD-1: program death 1; PDL-1: program death ligand 1; TCR: T cell receptor; MHC: major histocompatibility complex.

Although these findings of metabolic intervention were mostly based on cellular and animal models or early phase clinical trials as summarized in Table 2 and are yet unspecified on UCs, these encouraging results still offer a unique opportunity to restore host’s anti-tumor immunity by metabolic crosstalk in the TME. By combination of metabolic intervention with ICIs, we might have a chance to modulate the anti-tumor response not only through the antigen-dependent pathway but also influence the antigen-independent pathway. The mechanism of ICIs and metabolic intervention was summarized in Figure 1. Further research in metabolic intervention and TME is needed to break the present therapeutic stalemate on advanced and metastatic urothelial carcinoma and turn the metabolic intervention into a more specialized treatment pathway for urothelial carcinoma.

## 6. Conclusions

We introduced traditional chemotherapy of UC, followed by current evidence supporting the use of immune checkpoint inhibitors and ongoing clinical trials. Moreover, we reviewed the tumor metabolic characteristics and the anti-tumor treatments via metabolic pathways. A better strategy for UC management might be achieved by boosting T cell responses via ICIs and modulating cancer metabolism together. Further research in immunotherapy, including ICIs and immunometabolism intervention, is expected to provide new drug targets that modulate immune cells in a more selective way, providing more potent and less toxic choices for cancer management.

## Figures and Tables

**Table 1:** The indication and therapeutic regimen of programmed death 1 (PD-1) signal inhibitor approved by the U.S Food and Drug Administration (FDA).

	Indication	PD-1 Signal Inhibitor
First-line therapy	Cisplatin-ineligible or platinum-ineligible patients with PD-L1-positive advanced or metastatic UC	Atezolizumab
		Pembrolizumab
Second-line therapy	advanced or metastatic UC following failure of platinum-based therapy	Atezolizumab
		Avelumab
		Durvalumab
		Nivolumab
		Pembrolizumab
Maintenance therapy	local advanced or metastatic UC with objective response or stable disease after four to six cycles of GC regimen	Avelumab

UC: urothelial carcinoma; GC regimen: Gemcitabine plus cisplatin regimen.

**Table 2:** Clinical trial or experimental model on immune checkpoint inhibitor and metabolic intervention.

Pathway	Mechanism	Drugs	Trial(Phase)/Experimental Model	Result
Immune checkpoint inhibitor				
PD-1 pathway	blocking PD-L1 and PD-1 interaction	Atezolizumab	NCT02108652 (IMVigor210, phase II) NCT02302807 (IMVigor211, phase III)	FDA-approved first-line and second-line therapy of UC
		Avelumab	NCT01772004 (JAVELIN, phase III)	FDA-approved second-line and maintenance therapy of UC
		Durvalumab	NCT01693562 (Study 1108, phase I/II)	FDA-approved second-line therapy of UC
		Nivolumab	NCT01928394 (CheckMate 032, phase I/II) NCT02387996 (CheckMate-275, phase II)	FDA-approved second-line therapy of UC
		Pembrolizumab	NCT02256436 (KEYNOTE-045, phase III)	FDA-approved first-line and second-line therapy of UC
CTLA-4 pathway	blocking CTLA-4 and CD80/86 interaction	Tremelimumab	NCT02527434 (phase II)	comparable efficacy as PD-1/PD-L1 inhibitor, but more side effects
		Ipilimumab	-	-
Combination therapy of PD-1 and CTLA-4 pathway	blocking on PD-1 and CTLA-4 pathway	Nivolumab + Ipilimumab	NCT01928394 (CheckMate 032, phase I/II)	higher objective response rate to nivolumab monotherapy
			NCT03036098 (CheckMate 901, phase III)	ongoing
Metabolic intervention				
Glycolysis pathway	inhibit glycolysis via increasing deacetylase activity of SIRT6	Ellagic acid	Cellular model	anti-tumor effect
Lactate transport and metabolic	inhibit SLC16A1 and SLC16A7	AZ3965	NCT01791595 (phase I)	ongoing
		α-cyano-4-hydroxycinnamate	Cellular model	inhibit tumor proliferation and induce apoptosis
	LDHA inhibitor	Galloflavin	Cellular model	reduce cancer cells’ growth and induce apoptosis
Glutamine transport and metabolic	glutamine antagonist	6-diazo-5-oxo-L-norleucine	Cellular model	anti-tumor effect
	glutaminase inhibitor	CB-839	NCT03875313 (phase I/II)	ongoing

CTLA-4: cytotoxic T lymphocyte-associated antigen 4; FDA: U.S Food and Drug Administration; PD-1: programmed death 1; PD-L1: programmed death ligand 1; UC: urothelial carcinoma; LDHA: lactate dehydrogenase A.

## Abbreviations

UTUC	Upper-tract urothelial carcinoma
M-VAC	Methotrexate, Vinblastine, Doxorubicin, and Cisplatin
eGFR	Estimated glomerular filtration rate
CTLA-4	Cytotoxic T lymphocyte antigen 4
LDHA	Lactate dehydrogenase A
GLUT	Glucose transporters
GLUL	Glutamine synthetase
PD-L1	Programmed death ligand 1
UCB	Urothelial carcinoma of the bladder
APC	Antigen-presenting cell
PD-1	Programmed death 1
FDA	Food and Drug Administration
TME	Tumor microenvironment
UC	Urothelial carcinoma
AA	Aristolochic acid
ICI	Immune checkpoint inhibitor
GC	Gemcitabine plus cisplatin
NO	Nitrous oxide

## References

[1] Morales A., Eidinger D., Bruce A. (1976). Intracavitary Bacillus Calmette-guerin in the Treatment of Superficial Bladder Tumors. J. Urol..

[2] Kitamura H., Tsukamoto T. (2011). Immunotherapy for Urothelial Carcinoma: Current Status and Perspectives. Cancers.

[3] Yang H.-Y., Wu C.-Y., Powell J.D., Lu K.-L. (2020). Manipulation of Metabolic Pathways and Its Consequences for Anti-Tumor Immunity: A Clinical Perspective. Int. J. Mol. Sci..

[4] Siegel R.L., Miller K.D., Jemal A. (2019). Cancer statistics, 2019. CA Cancer J. Clin..

[5] Yang H.-Y., Yang C.-C., Wu C.-Y., Wang L.-J., Lu K.-L. (2019). Aristolochic Acid and Immunotherapy for Urothelial Carcinoma: Directions for unmet Needs. Int. J. Mol. Sci..

[6] Chen C.-H., Dickman K.G., Moriya M., Zavadil J., Sidorenko V.S., Edwards K.L., Gnatenko D.V., Wu L., Turesky R.J., Wu X.-R. (2012). Aristolochic acid-associated urothelial cancer in Taiwan. Proc. Natl. Acad. Sci. USA.

[7] Grollman A.P., Shibutani S., Moriya M., Miller F., Wu L., Moll U., Suzuki N., Fernandes A., Rosenquist T., Medverec Z. (2007). Aristolochic acid and the etiology of endemic (Balkan) nephropathy. Proc. Natl. Acad. Sci. USA.

[8] Von Der Maase H., Hansen S., Roberts J., Dogliotti L., Oliver T., Moore M., Bodrogi I., Albers P., Knuth A., Lippert C. (2000). Gemcitabine and Cisplatin Versus Methotrexate, Vinblastine, Doxorubicin, and Cisplatin in Advanced or Metastatic Bladder Cancer: Results of a Large, Randomized, Multinational, Multicenter, Phase III Study. J. Clin. Oncol..

[9] Hendricksen K., Witjes J.A. (2007). Current strategies for first and second line intravesical therapy for nonmuscle invasive bladder cancer. Curr. Opin. Urol..

[10] Steinbach F., Schuster F. (2005). Intravesical adjuvant chemotherapy for superficial bladder cancer—Results of a survey in Saxony. Aktuelle Urol..

[11] Kaufman D., Raghavan D., Carducci M., Levine E.G., Murphy B., Aisner J., Kuzel T., Nicol S., Oh W., Stadler W. (2000). Phase II Trial of Gemcitabine Plus Cisplatin in Patients With Metastatic Urothelial Cancer. J. Clin. Oncol..

[12] Lehmann J., Retz M., Steiner G., Albers P., Jaeger E., Knuth A., Lippert C., Koser M., Stockamp K., Otto C. (2003). Gemcitabine/cisplatin vs. MVAC. 5 year survival outcome of the phase III study of chemotherapy of advanced urothelial carcinoma in Germany. Urol. A.

[13] Wang Y., Xu L., Meng X., Qin Z., Wang Y., Chen C., Wang Y., Zhou X., Zhang Q., Xia J. (2018). Different Chemotherapy Regimens in the Management of Advanced or Metastatic Urothelial Cancer: A Bayesian Network Meta-Analysis of Randomized Controlled Trials. Cell. Physiol. Biochem..

[14] Bellmunt J., Mottet N., De Santis M. (2016). Urothelial carcinoma management in elderly or unfit patients. Eur. J. Cancer Suppl..

[15] Kim T.J., Cho K.S., Hong C.-H. (2020). Current Status and Future Perspectives of Immunotherapy for Locally Advanced or Metastatic Urothelial Carcinoma: A Comprehensive Review. Cancers.

[16] Thompson R.H., Boorjian S.A., Kim S.P., Cheville J.C., Thapa P., Tarrel R., Dronca R., Costello B., Frank I. (2013). Eligibility for neoadjuvant/adjuvant cisplatin-based chemotherapy among radical cystectomy patients. BJU Int..

[17] Dash A., Galsky M.D., Vickers A.J., Serio A.M., Koppie T.M., Dalbagni G., Bochner B.H. (2006). Impact of renal impairment on eligibility for adjuvant cisplatin-based chemotherapy in patients with urothelial carcinoma of the bladder. Cancer.

[18] Galsky M.D., Hahn N.M., Rosenberg J.E., Sonpavde G., Hutson T.E., Oh W.K., Dreicer R., Vogelzang N.J., Sternberg C.N., Bajorin D.F. (2011). Treatment of Patients With Metastatic Urothelial Cancer “Unfit” for Cisplatin-Based Chemotherapy. J. Clin. Oncol..

[19] Kaag M.G., O’Malley R.L., O’Malley P., Godoy G., Chen M., Smaldone M.C., Hrebinko R.L., Raman J.D., Bochner B., Dalbagni G. (2010). Changes in Renal Function Following Nephroureterectomy May Affect the Use of Perioperative Chemotherapy. Eur. Urol..

[20] Galsky M.D., Chen G.J., Oh W.K., Bellmunt J., Roth B.J., Petrioli R., Dogliotti L., Dreicer R., Sonpavde G. (2012). Comparative effectiveness of cisplatin-based and carboplatin-based chemotherapy for treatment of advanced urothelial carcinoma. Ann. Oncol..

[21] Small E.J., Fippin L.J., Ernest M.L., Carroll P.R. (1996). A carboplatin-based regimen for the treatment of patients with advanced transitional cell carcinoma of the urothelium. Cancer.

[22] Riera-Domingo C., Audigé A., Granja S., Cheng W.C., Ho P.C., Baltazar F., Stockmann C., Mazzone M. (2020). Immunity, Hypoxia, and Metabolism-the Menage a Trois of Cancer: Implications for Immunotherapy. Physiol. Rev..

[23] Koh J., Go H., Keam B., Kim M.-Y., Nam S.J., Kim T.M., Lee S.-H., Min H.S., Kim Y.T., Kim D.-W. (2015). Clinicopathologic analysis of programmed cell death-1 and programmed cell death-ligand 1 and 2 expressions in pulmonary adenocarcinoma: Comparison with histology and driver oncogenic alteration status. Mod. Pathol..

[24] Friedman C.F., Proverbs-Singh T.A., Postow M.A. (2016). Treatment of the Immune-Related Adverse Effects of Immune Checkpoint Inhibitors: A Review. JAMA Oncol..

[25] Thallinger C., Füreder T., Preusser M., Heller G., Müllauer L., Höller C., Prosch H., Frank N., Swierzewski R., Berger W. (2018). Review of cancer treatment with immune checkpoint inhibitors. Wien. Klin. Wochenschr..

[26] Thompson R.H., Kuntz S.M., Leibovich B.C., Dong H., Lohse C.M., Webster W.S., Sengupta S., Frank I., Parker A.S., Zincke H. (2006). Tumor B7-H1 Is Associated with Poor Prognosis in Renal Cell Carcinoma Patients with Long-term Follow-up. Cancer Res..

[27] Schenk-Braat E.A., Bangma C.H. (2005). Immunotherapy for superficial bladder cancer. Cancer Immunol. Immunother..

[28] Lim S.H., Sun J.-M., Lee S.-H., Ahn J.S., Park K., Ahn M.-J. (2016). Pembrolizumab for the treatment of non-small cell lung cancer. Expert Opin. Biol. Ther..

[29] Tran E., Ahmadzadeh M., Lu Y.-C., Gros A., Turcotte S., Robbins P.F., Gartner J.J., Zheng Z., Li Y.F., Ray S. (2015). Immunogenicity of somatic mutations in human gastrointestinal cancers. Science.

[30] Rosenberg J.E., Hoffman-Censits J., Powles T., Van Der Heijden M.S., Balar A.V., Necchi A., Dawson N., O’Donnell P.H., Balmanoukian A., Loriot Y. (2016). Atezolizumab in patients with locally advanced and metastatic urothelial carcinoma who have progressed following treatment with platinum-based chemotherapy: A single-arm, multicentre, phase 2 trial. Lancet.

[31] Kwon Y., Park M., Jang M., Yun S., Kim W.K., Kim S., Paik S., Lee H.J., Hong S., Kim T.I. (2017). Prognosis of stage III colorectal carcinomas with FOLFOX adjuvant chemotherapy can be predicted by molecular subtype. Oncotarget.

[32] Lawrence M.S., Stojanov P., Polak P., Kryukov G.V., Cibulskis K., Sivachenko A., Carter S.L., Stewart C., Mermel C.H., Roberts S.A. (2013). Mutational heterogeneity in cancer and the search for new cancer-associated genes. Nat. Cell Biol..

[33] Dorado B., Jerez M.J., Flores N., Martín-Saavedra F.M., Durán C., Ballester S. (2002). AutocrineIL-4Gene Regulation at Late Phases of TCR Activation in Differentiated Th2 Cells. J. Immunol..

[34] Iwai Y., Hamanishi J., Chamoto K., Honjo T. (2017). Cancer immunotherapies targeting the PD-1 signaling pathway. J. Biomed. Sci..

[35] Dolan D.E., Gupta S. (2014). PD-1 pathway inhibitors: Changing the landscape of cancer immunotherapy. Cancer Control..

[36] Brahmer J.R., Drake C.G., Wollner I., Powderly J.D., Picus J., Sharfman W.H., Stankevich E., Pons A., Salay T.M., McMiller T.L. (2010). Phase I study of single-agent anti-programmed death-1 (MDX-1106) in refractory solid tumors: Safety, clinical activity, pharmacodynamics, and immunologic correlates. J. Clin. Oncol..

[37] Torralba Cabeza M.A., Gonzalez S.O. (2017). Correlation study between genotype and phenotype: Success or failure?. Rev. Clin. Esp..

[38] Vuky J., Balar A.V., Castellano D., O’Donnell P.H., Grivas P., Bellmunt J., Powles T., Bajorin D., Hahn N.M., Savage M.J. (2020). Long-Term Outcomes in KEYNOTE-052: Phase II Study Investigating First-Line Pembrolizumab in Cisplatin-Ineligible Patients With Locally Advanced or Metastatic Urothelial Cancer. J. Clin. Oncol..

[39] Powles T., Duran I., van der Heijden M. (2018). Atezolizumab versus chemotherapy in patients with platinum-treated locally advanced or metastatic urothelial carcinoma (IMvigor211): A multicentre, open-label, phase 3 randomised controlled trial (vol 391, pg 748, 2018). Lancet.

[40] Bellmunt J., De Wit R., Vaughn D.J., Fradet Y., Lee J.-L., Fong L., Vogelzang N.J., Climent M.A., Petrylak D.P., Choueiri T.K. (2017). Pembrolizumab as Second-Line Therapy for Advanced Urothelial Carcinoma. N. Engl. J. Med..

[41] Sharma P., Callahan M.K., Bono P., Kim J., Spiliopoulou P., Calvo E., Pillai R.N., Ott P.A., De Braud F., Morse M. (2016). Nivolumab monotherapy in recurrent metastatic urothelial carcinoma (CheckMate 032): A multicentre, open-label, two-stage, multi-arm, phase 1/2 trial. Lancet Oncol..

[42] Sharma P., Retz M., Siefker-Radtke A., Baron A., Necchi A., Bedke J., Plimack E.R., Vaena D., Grimm M.-O., Bracarda S. (2017). Nivolumab in metastatic urothelial carcinoma after platinum therapy (CheckMate 275): A multicentre, single-arm, phase 2 trial. Lancet Oncol..

[43] Patel M.R., Ellerton J., Infante J.R., Agrawal M., Gordon M., Aljumaily R., Britten C.D., Dirix L., Lee K.-W., Taylor M. (2018). Avelumab in metastatic urothelial carcinoma after platinum failure (JAVELIN Solid Tumor): Pooled results from two expansion cohorts of an open-label, phase 1 trial. Lancet Oncol..

[44] Galluzzi L., Humeau J., Buqué A., Zitvogel L., Kroemer G. (2020). Immunostimulation with chemotherapy in the era of immune checkpoint inhibitors. Nat. Rev. Clin. Oncol..

[45] Galaine J., Turco C., Vauchy C., Royer B., Mercier-Letondal P., Queiroz L., Loyon R., Mouget V., Boidot R., Laheurte C. (2019). CD4 T cells target colorectal cancer antigens upregulated by oxaliplatin. Int. J. Cancer.

[46] Melief C.J.M., Kessler J.H. (2017). Novel insights into the HLA class I immunopeptidome and T-cell immunosurveillance. Genome Med..

[47] Pfirschke C., Engblom C., Rickelt S., Cortez-Retamozo V., Garris C., Pucci F., Yamazaki T., Poirier-Colame V., Newton A., Redouane Y. (2016). Immunogenic Chemotherapy Sensitizes Tumors to Checkpoint Blockade Therapy. Immunity.

[48] Yamazaki T., Buqué A., Ames T.D., Galluzzi L. (2020). PT-112 induces immunogenic cell death and synergizes with immune checkpoint blockers in mouse tumor models. OncoImmunology.

[49] Liu P., Zhao L., Pol J., Levesque S., Petrazzuolo A., Pfirschke C., Engblom C., Rickelt S., Yamazaki T., Iribarren K. (2019). Crizotinib-induced immunogenic cell death in non-small cell lung cancer. Nat. Commun..

[50] Ito T., Sato K., Oikawa M., Sugio H., Asanome T., Ozaki Y., Nakamura H., Tanaka K., Tsuda M., Nagashima K. (2015). Clinicopathological Study of Pilomyxoid-Spectrum Astrocytomas:An Analysis of the BRAF Gene. Report of Two Cases. No Shinkei Geka.

[51] Bailly C., Thuru X., Quesnel B. (2020). Combined cytotoxic chemotherapy and immunotherapy of cancer: Modern times. NAR Cancer.

[52] Seliger B. (2019). Combinatorial Approaches With Checkpoint Inhibitors to Enhance Anti-tumor Immunity. Front. Immunol..

[53] Shulman S.T. (2015). Changes Afoot, and the Pediatric SUPPORT Decision. Pediatr. Ann..

[54] Galsky M.D., Arija J.Á.A., Bamias A., Davis I.D., De Santis M., Kikuchi E., Garcia-Del-Muro X., De Giorgi U., Mencinger M., Izumi K. (2020). Atezolizumab with or without chemotherapy in metastatic urothelial cancer (IMvigor130): A multicentre, randomised, placebo-controlled phase 3 trial. Lancet.

[55] Eisele P., Drake K. (2020). Merck Provides Update on Phase 3 KEYNOTE-361 Trial Evaluating KEYTRUDA^®^ (pembrolizumab) as Monotherapy and in Combination with Chemotherapy in Patients with Advanced or Metastatic Urothelial Carcinoma, in Businesswire. https://www.bloomberg.com/press-releases/2020-06-09/merck-provides-update-on-phase-3-keynote-361-trial-evaluating-keytruda-pembrolizumab-as-monotherapy-and-in-combination-with.

[56] (2020). Avelumab Outduels Supportive Care for Urothelial Cancer. Cancer Discov..

[57] Powles T., Park S.H., Voog E., Caserta C., Valderrama B.P., Gurney H., Kalofonos H., Radulović S., Demey W., Ullén A. (2020). Avelumab Maintenance Therapy for Advanced or Metastatic Urothelial Carcinoma. N. Engl. J. Med..

[58] Rotte A. (2019). Combination of CTLA-4 and PD-1 blockers for treatment of cancer. J. Exp. Clin. Cancer Res..

[59] Carosella E.D., Ploussard G., LeMaoult J., Desgrandchamps F. (2015). A Systematic Review of Immunotherapy in Urologic Cancer: Evolving Roles for Targeting of CTLA-4, PD-1/PD-L1, and HLA-G. Eur. Urol..

[60] Chen M., Khadaroo P.A., Su H., Kong L., Chen L., Wang X., Li X., Zhu H., Zhong X., Pan J. (2019). The safety and tolerability of combined immune checkpoint inhibitors (anti-PD-1/PD-L1 plus anti-CTLA-4): A systematic review and meta-analysis. BMC Cancer.

[61] Leach D.R., Krummel M.F., Allison J.P. (1996). Enhancement of Antitumor Immunity by CTLA-4 Blockade. Science.

[62] Driessens G., Kline J., Gajewski T.F. (2009). Costimulatory and coinhibitory receptors in anti-tumor immunity. Immunol. Rev..

[63] Sharma P., Sohn J., Shin S.J., Oh D.-Y., Keam B., Lee H.J., Gizzi M., Kalinka E., De Vos F.Y., Ruscica D. (2020). Efficacy and Tolerability of Tremelimumab in Locally Advanced or Metastatic Urothelial Carcinoma Patients Who Have Failed First-Line Platinum-Based Chemotherapy. Clin. Cancer Res..

[64] (2019). Anti-CTLA-4 Antibody Tremelimumab Shows Efficacy in Patients with Metastatic Bladder Cancer. https://ecancer.org/en/news/17011-anti-ctla-4-antibody-tremelimumab-shows-efficacy-in-patients-with-metastatic-bladder-cancer.

[65] Bellmunt J., Powles T., Vogelzang N.J. (2017). A review on the evolution of PD-1/PD-L1 immunotherapy for bladder cancer: The future is now. Cancer Treat. Rev..

[66] Callahan M.K., Wolchok J.D. (2013). At the Bedside: CTLA-4- and PD-1-blocking antibodies in cancer immunotherapy. J. Leukoc. Biol..

[67] Chae Y.K., Arya A., Iams W., Cruz M.R., Chandra S., Choi J., Giles F. (2018). Current landscape and future of dual anti-CTLA4 and PD-1/PD-L1 blockade immunotherapy in cancer; lessons learned from clinical trials with melanoma and non-small cell lung cancer (NSCLC). J. Immunother. Cancer.

[68] Macdonald D., Aston M., Murphy G.T., Jefferies K., Mselle L.T., Price S.L., O’Hearn S., White M., Mbekenga C.K., Kohi T.W. (2018). Providing postpartum care with limited resources: Experiences of nurse-midwives and obstetricians in urban Tanzania. Women Birth.

[69] Michot J.-M., Bigenwald C., Champiat S., Collins M., Carbonnel F., Postel-Vinay S., Berdelou A., Varga A., Bahleda R., Hollebecque A. (2016). Immune-related adverse events with immune checkpoint blockade: A comprehensive review. Eur. J. Cancer.

[70] De Velasco G., Je Y., Bossé D., Awad M.M., Ott P.A., Moreira R.B., Schutz F., Bellmunt J., Sonpavde G.P., Hodi F.S. (2017). Comprehensive Meta-analysis of Key Immune-Related Adverse Events from CTLA-4 and PD-1/PD-L1 Inhibitors in Cancer Patients. Cancer Immunol. Res..

[71] Hellmann M.D., Friedman C.F., Wolchok J.D. (2016). Combinatorial Cancer Immunotherapies. Adv. Immunol..

[72] Topalian S.L., Drake C.G., Pardoll D.M. (2015). Immune Checkpoint Blockade: A Common Denominator Approach to Cancer Therapy. Cancer Cell.

[73] Spranger S., Luke J.J., Bao R., Zha Y., Hernandez K.M., Li Y., Gajewski A.P., Andrade J., Gajewski T.F. (2016). Density of immunogenic antigens does not explain the presence or absence of the T-cell–inflamed tumor microenvironment in melanoma. Proc. Natl. Acad. Sci. USA.

[74] Lanitis E., Dangaj D., Irving M., Coukos G. (2017). Mechanisms regulating T-cell infiltration and activity in solid tumors. Ann. Oncol..

[75] Bonaventura P., Shekarian T., Alcazer V., Valladeau-Guilemond J., Valsesia-Wittmann S., Amigorena S., Caux C., Depil S. (2019). Cold Tumors: A Therapeutic Challenge for Immunotherapy. Front. Immunol..

[76] Kather J.N., Suarez-Carmona M., Charoentong P., Weis C.-A., Hirsch D., Bankhead P., Horning M., Ferber D., Kel I., Herpel E. (2018). Topography of cancer-associated immune cells in human solid tumors. eLife.

[77] Whiteside T.L. (2008). The tumor microenvironment and its role in promoting tumor growth. Oncogene.

[78] Baghban R., Roshangar L., Jahanban-Esfahlan R., Seidi K., Ebrahimi-Kalan A., Jaymand M., Kolahian S., Javaheri T., Zare P. (2020). Tumor microenvironment complexity and therapeutic implications at a glance. Cell Commun. Signal..

[79] Francoa P.I.R., Rodrigues A.P., De Menezes L.B., Miguel M.P. (2020). Tumor microenvironment components: Allies of cancer progression. Pathol. Res. Pract..

[80] Iovanna J.L., Closa D. (2017). Factors released by the tumor far microenvironment are decisive for pancreatic adenocarcinoma development and progression. OncoImmunology.

[81] Adekola K., Rosen S.T., Shanmugam M. (2012). Glucose transporters in cancer metabolism. Curr. Opin. Oncol..

[82] Liberti M.V., Locasale J.W. (2016). The Warburg Effect: How Does it Benefit Cancer Cells?. Trends Biochem. Sci..

[83] Schwartz L., Supuran C.T., Alfarouk K.O. (2017). The Warburg Effect and the Hallmarks of Cancer. Anti-Cancer Agents Med. Chem..

[84] Xu X.D., Shao S.X., Jiang H.P., Cao Y.W., Wang Y.H., Yang X.C., Wang Y.L., Wang X.S., Niu H.T. (2015). Warburg Effect or Reverse Warburg Effect? A Review of Cancer Metabolism. Oncol. Res. Treat..

[85] Sebastián C., Zwaans B.M.M., Silberman D.M., Gymrek M., Goren A., Zhong L., Ram O., Truelove J., Guimaraes A.R., Toiber D. (2012). The Histone Deacetylase SIRT6 Is a Tumor Suppressor that Controls Cancer Metabolism. Cell.

[86] Zhang G., Liu Z., Qin S., Li K. (2015). Decreased expression of SIRT6 promotes tumor cell growth correlates closely with poor prognosis of ovarian cancer. Eur. J. Gynaecol. Oncol..

[87] Teixeira M., Sánchez-López E., Espina M., García M.L., Durazzo A., Lucarini M., Novellino E., Souto S.B., Santini A., Souto E.B. (2019). Sirtuins and SIRT6 in Carcinogenesis and in Diet. Int. J. Mol. Sci..

[88] Tian J., Yuan L. (2018). Sirtuin 6 inhibits colon cancer progression by modulating PTEN/AKT signaling. Biomed. Pharmacother..

[89] Hu J., Deng F., Hu X., Zhang W., Zeng X., Tian X. (2018). Histone deacetylase SIRT6 regulates chemosensitivity in liver cancer cells via modulation of FOXO3 activity. Oncol. Rep..

[90] Garcia-Peterson L.M., Guzmán-Pérez G., Krier C.R., Ahmad N. (2020). The sirtuin 6: An overture in skin cancer. Exp. Dermatol..

[91] Rahnasto-Rilla M., Järvenpää J., Huovinen M., Schroderus A.-M., Ihantola E.-L., Küblbeck J., Khadeer M., Moaddel R., Lahtela-Kakkonen M. (2020). Effects of galloflavin and ellagic acid on sirtuin 6 and its anti-tumorigenic activities. Biomed. Pharmacother..

[92] Ahire V., Kumar A., Mishra K., Kulkarni G. (2017). Ellagic Acid Enhances Apoptotic Sensitivity of Breast Cancer Cells to γ-Radiation. Nutr. Cancer.

[93] Chen H.-S., Bai M.-H., Zhang T., Li G.-D., Liu C.-M. (2015). Ellagic acid induces cell cycle arrest and apoptosis through TGF-?/Smad3 signaling pathway in human breast cancer MCF-7 cells. Int. J. Oncol..

[94] Romero-Garcia S., Moreno-Altamirano M.M.B., Prado-Garcia H., Sánchez-García F.J. (2016). Lactate Contribution to the Tumor Microenvironment: Mechanisms, Effects on Immune Cells and Therapeutic Relevance. Front. Immunol..

[95] Rattigan Y.I., Patel B.B., Ackerstaff E., Sukenick G., Koutcher J.A., Glod J.W., Banerjee D. (2012). Lactate is a mediator of metabolic cooperation between stromal carcinoma associated fibroblasts and glycolytic tumor cells in the tumor microenvironment. Exp. Cell Res..

[96] Busk M., Walenta S., Mueller-Klieser W., Steiniche T., Jakobsen S., Horsman M.R., Overgaard J. (2011). Inhibition of tumor lactate oxidation: Consequences for the tumor microenvironment. Radiother. Oncol..

[97] Mishra D., Banerjee D. (2019). Lactate Dehydrogenases as Metabolic Links between Tumor and Stroma in the Tumor Microenvironment. Cancers.

[98] Bellone M., Calcinotto A., Filipazzi P., De Milito A., Fais S., Rivoltini L. (2013). The acidity of the tumor microenvironment is a mechanism of immune escape that can be overcome by proton pump inhibitors. OncoImmunology.

[99] Nakagawa Y., Negishi Y., Shimizu M., Takahashi M., Ichikawa M., Takahashi H. (2015). Effects of extracellular pH and hypoxia on the function and development of antigen-specific cytotoxic T lymphocytes. Immunol. Lett..

[100] Polański R., Hodgkinson C.L., Fusi A., Nonaka D., Priest L., Kelly P., Trapani F., Bishop P.W., White A., Critchlow S.E. (2014). Activity of the Monocarboxylate Transporter 1 Inhibitor AZD3965 in Small Cell Lung Cancer. Clin. Cancer Res..

[101] Mathupala S.P., Parajuli P., Sloan A.E. (2004). Silencing of monocarboxylate transporters via small interfering ribonucleic acid inhibits glycolysis and induces cell death in malignant glioma: An in vitro study. Neurosurgery.

[102] Brand A., Singer K., Koehl G.E., Kolitzus M., Schoenhammer G., Thiel A., Matos C., Bruss C., Klobuch S., Peter K. (2016). LDHA-Associated Lactic Acid Production Blunts Tumor Immunosurveillance by T and NK Cells. Cell Metab..

[103] Granchi C., Paterni I., Rani R., Minutolo F. (2013). Small-molecule inhibitors of human LDH5. Futur. Med. Chem..

[104] Farabegoli F., Vettraino M., Manerba M., Fiume L., Roberti M., Di Stefano G. (2012). Galloflavin, a new lactate dehydrogenase inhibitor, induces the death of human breast cancer cells with different glycolytic attitude by affecting distinct signaling pathways. Eur. J. Pharm. Sci..

[105] Cluntun A.A., Lukey M.J., Cerione R.A., Locasale J.W. (2017). Glutamine Metabolism in Cancer: Understanding the Heterogeneity. Trends Cancer.

[106] Yang L., Achreja A., Yeung T.-L., Mangala L.S., Jiang D., Han C., Baddour J., Marini J.C., Ni J., Nakahara R. (2016). Targeting Stromal Glutamine Synthetase in Tumors Disrupts Tumor Microenvironment-Regulated Cancer Cell Growth. Cell Metab..

[107] Li T., Le A. (2018). Glutamine Metabolism in Cancer. Adv. Exp. Med. Biol..

[108] Issaq S.H., Mendoza A., Fox S.D., Helman L.J. (2019). Glutamine synthetase is necessary for sarcoma adaptation to glutamine deprivation and tumor growth. Oncogenesis.

[109] Cruzat V., Macedo Rogero M., Noel Keane K., Curi R., Newsholme P. (2018). Glutamine: Metabolism and Immune Function, Supplementation and Clinical Translation. Nutrients.

[110] Duran R.V., Hall M.N. (2012). Glutaminolysis feeds mTORC1. Cell Cycle.

[111] Csibi A., Fendt S.-M., Li C., Poulogiannis G., Choo A.Y., Chapski D.J., Jeong S.M., Dempsey J.M., Parkhitko A., Morrison T. (2013). The mTORC1 Pathway Stimulates Glutamine Metabolism and Cell Proliferation by Repressing SIRT4. Cell.

[112] Feng M., Xiong G., Cao Z., Yang G., Zheng S., Qiu J., You L., Zheng L., Zhang T., Zhao Y. (2018). LAT2 regulates glutamine-dependent mTOR activation to promote glycolysis and chemoresistance in pancreatic cancer. J. Exp. Clin. Cancer Res..

[113] Cervantes-Madrid D., Romero Y., Dueñas-González A. (2015). Reviving Lonidamine and 6-Diazo-5-oxo-L-norleucine to Be Used in Combination for Metabolic Cancer Therapy. BioMed Res. Int..

[114] Garber K.G.K. (2016). Cancer anabolic metabolism inhibitors move into clinic. Nat. Biotechnol..

[115] Gross M.I., Demo S.D., Dennison J.B., Chen L., Chernov-Rogan T., Goyal B., Janes J.R., Laidig G.J., Lewis E.R., Li J. (2014). Antitumor Activity of the Glutaminase Inhibitor CB-839 in Triple-Negative Breast Cancer. Mol. Cancer Ther..

[116] Boysen G., Jamshidi-Parsian A., Davis M.A., Siegel E.R., Simecka C.M., Kore R.A., Dings R.P.M., Griffin R.J. (2019). Glutaminase inhibitor CB-839 increases radiation sensitivity of lung tumor cells and human lung tumor xenografts in mice. Int. J. Radiat. Biol..

